# White matter pathology in Parkinson's disease: The effect of imaging protocol differences and relevance to executive function

**DOI:** 10.1016/j.neuroimage.2012.06.012

**Published:** 2012-09

**Authors:** Charlotte L. Rae, Marta M. Correia, Ellemarije Altena, Laura E. Hughes, Roger A. Barker, James B. Rowe

**Affiliations:** a MRC Cognition and Brain Sciences Unit, Cambridge, CB2 7EF, UK; b Department of Clinical Neurosciences, University of Cambridge, Cambridge, CB2 2QQ, UK; c Cambridge Centre for Brain Repair, University of Cambridge, CB2 0PY, UK; d Behavioural and Clinical Neuroscience Institute, University of Cambridge, Cambridge, CB2 3EB, UK

**Keywords:** DTI, diffusion tensor imaging, FA, fractional anisotropy, FWE, family-wise error, H&Y, Hoehn and Yahr, MD, mean diffusivity, MMSE, mini-mental state examination, MR, magnetic resonance, PD, Parkinson's disease, TBSS, tract-based spatial statistics, TFCE, threshold-free cluster enhancement, UPDRS, Unified Parkinson's Disease Rating Scale, DTI, TBSS, VBM, Parkinson's disease, Executive function

## Abstract

Diffusion magnetic resonance imaging is increasingly used as a non-invasive method to investigate white matter structure in neurological and neuropsychiatric disease. However, many options are available for the acquisition sequence and analysis method. Here we used Parkinson's disease as a model neurodegenerative disorder to compare imaging protocols and analysis options. We investigated fractional anisotropy and mean diffusivity of white matter in patients and age-matched controls, comparing two datasets acquired with different imaging protocols. One protocol prioritised the number of *b* value acquisitions, whilst the other prioritised the number of gradient directions. The dataset with more gradient directions was more sensitive to reductions in fractional anisotropy in Parkinson's disease, whilst the dataset with more *b* values was more sensitive to increases in mean diffusivity. Moreover, the areas of reduced fractional anisotropy were highly similar to areas of increased mean diffusivity in PD patients. Next, we compared two widely used analysis methods: tract-based spatial statistics identified reduced fractional anisotropy and increased mean diffusivity in Parkinson's disease in many of the major white matter tracts in the frontal and parietal lobes. Voxel-based analyses were less sensitive, with similar patterns of white matter pathology observed only at liberal statistical thresholds. We also used tract-based spatial statistics to identify correlations between a test of executive function (phonemic fluency), fractional anisotropy and mean diffusivity in prefrontal white matter in both Parkinson's disease patients and controls. These findings suggest that in Parkinson's disease there is widespread pathology of cerebral white matter, and furthermore, pathological white matter in the frontal lobe may be associated with executive dysfunction. Diffusion imaging protocols that prioritised the number of directions versus the number of *b* values were differentially sensitive to alternative markers of white matter pathology, such as fractional anisotropy and mean diffusivity.

## Highlights

► DTI shows abnormal frontal white matter in Parkinson's disease. ► FA and MD in the frontal lobe correlate with executive function in PD. ► The number of directions and *b* values affects sensitivity to FA/MD changes in PD. ► TBSS is more sensitive than VBM to white matter change in Parkinson's disease.

## Introduction

Although Parkinson's disease (PD) is defined by its motor syndrome – akinesia, resting tremor, and rigidity – it is also associated with many non-motor symptoms and cognitive deficits (Chaudhuri and Schapira, 2009; Kehagia et al., 2010). The diversity of these motor and cognitive phenomena reflects the widespread progression of underlying pathology, including alphasynuclein-based Lewy body aggregates, loss of dopaminergic projections from the substania nigra to the striatum, and progressive loss of cholinergic and monoaminergic cortical projections from the forebrain (Braak et al., 2006; Spillantini et al., 1997). Grey matter atrophy occurs in cortical and subcortical regions (Jubault et al., 2009; Lee et al., 2010a; Nishio et al., 2010; Pereira et al., in press; Reetz et al., 2009; Rowe et al., 2010; Song et al., 2011). There are also pathological changes in white matter in the form of Lewy neurites, which accumulate in brainstem axons and later, in the cerebral white matter (Braak et al., 2006). However, the extent of white matter pathology in PD, and its impact upon motor and cognitive function, remains uncertain.

Diffusion tensor imaging (DTI) is an effective non-invasive tool to investigate pathological changes in the white matter of living neurodegenerative and psychiatric patients (Acosta-Cabronero et al., 2010; Douaud et al., 2011; Kubicki and Shenton, 2009; Peran et al., 2010; Wang et al., 2009). DTI is sensitive to the diffusion of water molecules within neural tissue (Basser et al., 1994). Water molecules tend to diffuse longitudinally along an axonal trajectory, rather than in the perpendicular direction, due to hindrance from the axonal myelin sheath, the cell membrane, and intracellular structures. Scalar measures of diffusion thereby act as markers for white matter tract microstructure. Two measures commonly used to assess tract microstructure in health (Scholz et al., 2009) and in disease (Bodini and Ciccarelli, 2009) are fractional anisotropy (FA) and mean diffusivity (MD). FA characterises diffusion in the axonal orientation relative to two orthogonal radial orientations, representing how strongly directional diffusion is within white matter. MD characterises the mean diffusion along the axonal and radial orientations, representing how restricted diffusion is within white matter. Reductions in FA or increases in MD are therefore often used as markers of a change in myelination or degradation of axonal structure.

Previous DTI studies have used FA and MD to investigate white matter microstructure in PD and related disorders (such as PD dementia and dementia with Lewy bodies). Many have taken a region-of-interest approach in which structures of interest are manually defined on MR images (Chan et al., 2007; Gattellaro et al., 2009; Modrego et al., 2011; Peran et al., 2010; Rolheiser et al., 2011; Vaillancourt et al., 2009). Several of these region-of-interest studies have identified reduced FA or increased MD in areas of a priori relevance to PD, such as the substantia nigra. However, manual region-of-interest comparison of diffusion measures is subject to inter-rater variability, and moreover, limits the regions in which significant group differences can be found.

Whole-brain analysis methods permit group comparisons of diffusion measures outside specified regions-of-interest. DTI studies of PD have used voxel-based analyses (Karagulle Kendi et al., 2008; Lee et al., 2010b; Zhan et al., 2012; Zhang et al., 2011) and skeleton-based analyses (Hattori et al., 2012; Ibarretxe-Bilbao et al., 2010). These studies have identified reduced FA in PD in regions outside the substantia nigra that have been putatively associated with Lewy neurite pathology, such as the olfactory tract (Ibarretxe-Bilbao et al., 2010; Zhang et al., 2011), external capsule (Zhan et al., 2012), and prefrontal white matter (Karagulle Kendi et al., 2008).

However, key questions remain. What is the optimum diffusion imaging protocol when imaging PD patients? Given limited imaging time in many clinical and research settings, a briefer diffusion sequence is advantageous with regards to both patients' comfort and data quality (as subjects will move less). It is therefore important to identify a short diffusion imaging protocol that is sensitive to group differences in diffusion measures between PD patients and controls. The possibility of large motion artefacts, either from a resting tremor or sudden movements to relieve akinetic-rigidity, should also be considered. A diffusion imaging protocol incorporating both multiple *b* value acquisitions and a large number of gradient directions can improve the accuracy of diffusion measures such as FA and MD (Correia et al., 2009). However, when time is limited, a diffusion imaging protocol must prioritise one or the other. A larger number of *b* values per direction can improve the estimation of diffusivity along each direction, particularly in the presence of lower signal to noise (which could be the case for example, if PD patients had greater motion artefacts). In contrast, a larger number of gradient directions enable more accurate spatial reconstruction of the white matter structure through greater angular resolution. We therefore acquired two DTI datasets from the same PD patients and controls, each with a diffusion imaging protocol prioritising either *b* values or gradient directions, to compare the sensitivity of the two protocols with regards to detection of group differences in diffusion measures.

A second important consideration is the method of analysis of DTI data. Although whole-brain studies of diffusion measures in PD have utilised either a voxel- or skeleton-based approach, none of these PD studies has examined the benefits of one approach over the other. Several issues have been raised regarding the validity of a voxel-based approach for DTI data (Smith and Kindlmann, 2009). In particular, one cannot be certain that the same region of white matter tract corresponds accurately across subjects. The tract-based spatial statistics (TBSS) method aims to solve this correspondence problem by creation of a “mean FA skeleton”, onto which individual subject FA images are projected before voxelwise statistics are performed (Smith et al., 2006). Whilst maintaining control of tract localisation is important, we also aimed to determine which of the two methods would be more sensitive to FA or MD alterations in PD; we therefore repeated the analysis of our two datasets using both TBSS and voxel-based methods.

Structure–function correlations with diffusion measures can be useful to characterise individual differences and heterogeneity of disease. In the specific case of PD, the significance of white matter tract microstructure alterations for motor and cognitive function disease remains unknown. A patient's score on section III of the Unified Parkinson's Disease Rating Scale (UPDRS; Fahn et al., 1987) is commonly used to indicate the level of motor disability. Two studies have identified correlations between UPDRS scores and FA in the substantia nigra (Modrego et al., 2011; Zhan et al., 2012). However, no associations were found between white matter tract microstructure and motor function. With regards to cognitive function, Hattori et al. (2012) correlated mini-mental state examination (MMSE) scores with FA measures in PD patients. Patients with cognitive impairment showed a correlation between FA in parietal white matter and MMSE scores. No correlation was identified, however, between FA and MMSE scores for patients with “normal cognition”. Nonetheless, PD patients with apparently normal cognition (i.e. with a MMSE score that falls within the normal range of ≥ 26), may still show specific deficits in executive function (Kehagia et al., 2010; Williams-Gray et al., 2007). For example, phonemic fluency can be impaired in PD, including in non-demented patients (Mimura et al., 2006; Williams-Gray et al., 2007; Witt et al., 2006). Therefore we correlated diffusion measures with motor and executive function in our non-demented sample of PD patients, using UPDRS and phonemic fluency scores.

This study therefore sought to address the following questions: when collecting diffusion imaging data and time is limited, which acquisition parameters should be prioritised? Are voxel-based or skeleton-based analyses more sensitive to group differences in FA and MD? What is the extent of white matter pathology in early to mid stage PD? Finally, is such white matter pathology related to patients' motor and cognitive function?

## Methods

### Subjects

Fourteen patients with idiopathic PD were recruited from the Cambridge Centre for Brain Repair's PD research clinic, and met the criteria for diagnosis of Parkinson's disease according to the UK PD Brain Bank criteria (Hughes et al., 1992). Patients were in an early to mid stage of disease, according to the Hoehn and Yahr system (H&Y, Hoehn and Yahr, 1967) (stages 1.5 to 3, none in stage 4 or 5). A neurologist experienced in movement disorders (JBR) undertook the UPDRS-III motor subscale and H&Y rating scale prior to scanning on all patients. Patients were not demented, based on the clinician review at their most recent clinic assessment and corroborated by a within-study score of 26/30 or more on the mini-mental state examination (MMSE, Folstein et al., 1975). Fifteen neurologically healthy age-matched controls were recruited from the Medical Research Council Cognition and Brain Sciences Unit volunteer panel. All subjects undertook a phonemic fluency test (number of words beginning with ‘p’ in 1 min) (Benton, 1968). See Table 1 for subject demographic and neuropsychological evaluation information, and patient disease severity information. Details of patients' medications and doses are contained within the Supplementary information. All subjects gave informed written consent in accordance with the Declaration of Helsinki. The study was approved by the Local Research Ethics Committee.

### Diffusion magnetic resonance image acquisition and pre-processing

Two sets of diffusion-weighted data were collected from subjects on separate scanning days, as part of a study in which patients were scanned once whilst taking their normal dopaminergic medication, and once after medication delay. The two diffusion datasets were collected an average of 22 days (3 weeks) apart (minimum between scan duration: 11 days, maximum: 42 days), and were counterbalanced for patients being on- or off-medication. On both scanning sessions, twice refocused spin echo diffusion-weighted images were acquired on a Siemens Trio 3T, with a voxel size of 2 × 2 × 2 mm³ (2 mm interleaved axial slices; matrix size 96 × 96; field of view 192 × 192 mm).

In one dataset (“12 × 5”), diffusion-weighting was applied along 12 gradient directions, with 5 acquisitions, one acquisition at each of the following *b* values: 250 s/mm², 500 s/mm, 750 s/mm², 950 s/mm², and 1200 s/mm². 6 images with no diffusion-weighting (*b* = 0 s/mm²) were acquired throughout the sequence. In the second dataset (“30 × 2”), diffusion-weighting was applied along 30 gradient directions, with 2 acquisitions, one at a *b* value of 600 s/mm², and the other at 1200 s/mm². 5 images with no diffusion-weighting (*b* = 0 s/mm²) were acquired throughout the sequence. For both protocols, the gradient directions were optimised according to criteria described by Jones et al. (1999). Each diffusion imaging protocol took approximately 10 min.

Data were preprocessed and analysed using FMRIB Software Library, version 4.1 (FSL; www.fmrib.ox.ac.uk/fsl). Diffusion-weighted images were converted from dicom to nifti format, and corrected for eddy currents and subject motion by affine registration to the first *b*0 image using the FSL “eddy_correct” function. Each subject's movement in x, y and z co-ordinates was calculated according to the output of eddy_correct. Diffusion tensors were linearly fitted to the diffusion-weighted images using the FSL tool “dtifit”, giving output maps of FA, MD, AD (axial diffusivity) and RD (radial diffusivity).

To determine whether PD patients' movements during image acquisition were greater than controls', a measure of volume-to-volume displacement was obtained for each subject using the output logfile of the “eddy_correct” motion correction function. From the “eddy_correct” logfile, we took the translation measurement in x, y, and z co-ordinates between each volume, and then calculated the root mean square of the three translations. We summed this measure across all the volumes to give an index of the total displacement translations (the path length in three dimensions) throughout the sequence acquisition. This index was taken as a measure of subject motion during the 10 min diffusion sequence. Rotations and translations are highly correlated during scanning, and therefore we did not include a rotation factor in our summary metric of movement. A repeated measures analysis of variance with a within-subjects factor of imaging protocol (12 × 5 or 30 × 2) and between-subjects factor of subject group (PD patient or control) indicated whether PD patients had significantly worse motion than control subjects.

### Tract-based spatial statistics

The FSL tool “TBSS” (Tract-based spatial statistics) (Smith et al., 2006) was used to compare diffusion measures between PD patients and controls, to correlate diffusion measures with motor and cognitive function, and to compare between the two datasets acquired using the different diffusion imaging protocols. Subjects' individual FA images were first registered to a common template. As PD is a neurodegenerative disease, some degree of atrophy is often present, which can result in enlarged ventricles. With normal ageing, there can also be atrophy and enlargement of the ventricles. Registration to a study-specific image was chosen with the aim of more accurate alignment of the centre of tracts across subjects. Subjects' FA images were therefore registered to the FA image of the most “representative” subject, which is automatically detected by TBSS according to which image requires the least transformation to the FA images of all other subjects (in this instance, the FA image of a control from the 30 × 2 dataset). After subjects' individual FA images were registered to this initial template, they were registered to MNI152 space for convenience of display and reporting. The mean FA skeleton, a representation of the centre of the white matter tracts common to all subjects, was created and thresholded at FA > 0.25. Subjects' MNI152-registered FA images were projected onto the skeleton before statistics were performed on each skeleton voxel using permutations testing with the FSL tool “randomise”. A similar process was performed for the MD, AD, and RD images, without the initial registrations: the MD, AD, and RD images were projected onto the same skeleton, and statistics performed with randomise.

A design matrix was generated using the FSL tool “Glm”, in which images were categorised according to subject group (PD patient or control) and imaging protocol (12 × 5 or 30 × 2). The repeated measures element of the design was specified using the “group” option in the Glm tool. White matter microstructure changes with age (Salat et al., 2009). In this study, a primary aim was to search for evidence of white matter pathology related to PD, and not due to age-related change. Subject age was therefore entered into the design matrix as a confounding covariate, for each group separately. Gender was additionally entered as a confounding covariate for the two groups together (any gender difference in white matter microstructure is assumed to be similar in PD patients and controls). UPDRS scores off-medication were entered as a covariate of interest, to establish if white matter pathology correlates with severity of motor symptoms. Phonemic fluency scores for both PD patients and controls were also entered as a covariate of interest, to investigate whether white matter microstructure correlates with executive function. All covariates except gender were demeaned before values were entered.

This design matrix was used to analyse subjects' FA, MD, AD, and RD images using the FSL tool randomise, with 5000 permutations. The randomise option threshold-free cluster enhancement (TFCE, Smith and Nichols, 2009) was applied. All statistic images were corrected for multiple comparisons and thresholded at *p* < 0.01. Any regions on the skeleton showing significant clusters were localised using the “John Hopkins University ICBM-DTI-81 White Matter Labels” and “John Hopkins University White Matter Tractography” atlases in FSL.

To validate accurate registration to the study-specific template in the first step of TBSS, the TBSS function “deproject” was used to transform any significant statistic images generated by the voxelwise statistics back to native subject space. There were no significant voxels in any of the contrasts that showed deviation from white matter when deprojected back to native subject space, thus suggesting that any significant findings cannot be attributed to mis-registration. See Supplementary information for images from a sample significant statistic image (FA C > PD 30 × 2; the statistic with the largest number of significant voxels) deprojected back into each subject's native space.

### Voxel-based analyses

A study-specific FA template was created using the FA images from all subjects (Abe et al., 2010). First, all subjects' FA images were coregistered to the standard space FA template provided by FSL (FMRIB58_1mm) using an affine 12-parameter transformation, followed by a non-linear transformation. The resulting normalised FA images were then smoothed with an 8 mm isotropic Gaussian kernel, and a mean image (FA template) was created. Individual subjects' FA images were then registered to the customised FA template using FSL registration tools.

All subsequent steps for voxel-based analysis were performed using the SPM8 package (www.fil.ion.ucl.ac.uk/spm). The transformed FA images were smoothed with Gaussian kernels with full width half maximum of 4 mm. A general linear model included subject group (PD patient or control) as a factor, plus age and gender as confounding variables, and UPDRS scores and phonemic fluency as covariates of interest (thus resembling the TBSS model above). Statistically significant differences in FA and MD between PD patients and controls were assessed with a repeated measures analysis of variance. In addition, all tests on FA were performed using an absolute threshold of FA > 0.2, such that if a voxel had an FA value of less than 0.2 in any one subject, that voxel was not considered for analysis. These thresholds help to minimise the comparison of different structures in different subjects and to constrain the total number of voxels to be analysed. An initial threshold *p*-value of < 0.01 (one-tailed) corrected with familywise error (FWE) rate and a cluster size greater than 50 voxels was considered to be statistically significant. An exploratory threshold (*p* < 0.001 uncorrected) is also reported.

## Results

### Neuropsychological scores

PD patients were well matched to controls for age and gender (Table 1). They had normal scores on the mini-mental state examination (*t* = 1.52, *p* = 0.14, mean PD = 28.36, mean control = 29; no subjects with a score below 26). Their phonemic fluency scores did not differ from controls' (*t* = 0.24, *p* = 0.81, mean PD = 14.57, mean control = 15).

### Subject motion

In a repeated measures analysis of variance, there was no significant main effect of group (PD patient or control) on total amount of movement during scanning (*F* = 0.05, *p* = 0.82). There was no significant interaction between group and imaging protocol (*F* = 0.61, *p* = 0.44). Thus the total amount of subject movement throughout the two sequence acquisitions did not differ between PD patients and controls. The main effect of imaging protocol was also not significant (*F* = 0.108, *p* = 0.744), indicating that motion artefacts were no worse during one imaging protocol than the other. See Supplementary information for mean total amount of subject movement by PD patients and controls during each of the two imaging sequences.

### Tract-based spatial statistics

**Fig. 1:**
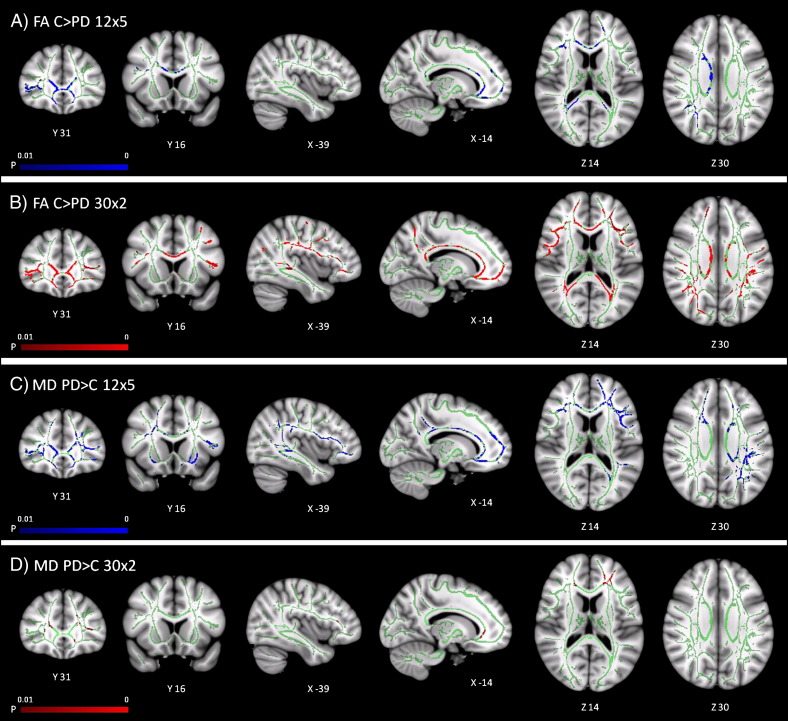
TBSS: regions of reduced FA (*p* < 0.01 corrected) in PD patients in A) the 12 × 5 dataset, and B) 30 × 2 dataset, and increased MD (*p* < 0.01 corrected) in C) the 12 × 5 and D) 30 × 2 datasets. TBSS results are shown overlaid on an MNI152 template and the mean FA skeleton (green).

PD patients had reduced FA, and increased MD, in widespread regions of cerebral white matter (see Fig. 1). However, there were differences in the extent of the reductions in FA and increases in MD in the two datasets acquired with different diffusion imaging protocols. Both the 12 × 5 and 30 × 2 datasets identified reduced FA (*p* < 0.01) in PD patients in prefrontal and parietal white matter, the corpus callosum, and the superior corticospinal tract; both datasets identified increased MD (*p* < 0.01) in prefrontal white matter and the corpus callosum.

However, the 30 × 2 dataset identified a much larger number of voxels showing a reduction in FA in PD patients in prefrontal and parietal white matter, the corpus callosum, and superior corticospinal tract than the 12 × 5 dataset. Additionally, the 30 × 2 dataset also identified reductions in FA in temporal lobe white matter. The 12 × 5 dataset, in contrast, identified increases in MD (*p* < 0.01) in parietal and temporal lobe white matter, in the left mid- and superior corticospinal tract, and the internal and external capsules, changes that were not found in the 30 × 2 dataset. Table 2 lists all tracts identified as having reduced FA or increased MD in PD patients in each dataset. There were increases in AD and RD in many of the regions showing reductions in FA and increases in MD (see Fig. S2 in Supplementary information). There were no regions of increased FA or decreased MD (*p* < 0.01) in PD patients in either of the two datasets.

Although the 30 × 2 dataset identified more widespread reductions in FA in PD patients, and the 12 × 5 dataset more widespread increases in MD, the interaction between subject group and imaging protocol did not reveal any significant differences between protocols, for either FA or MD (*p* < 0.01). Thus although the 30 × 2 sequence was more sensitive to group differences in FA, and the 12 × 5 sequence more sensitive to differences in MD, they were not significantly different from each other in a direct comparison.

### Motor severity

**Fig. 2:**
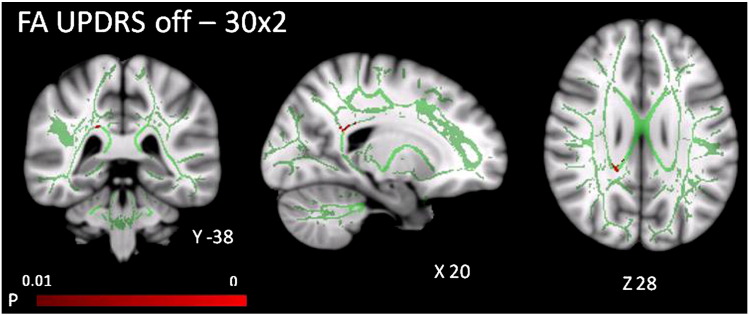
TBSS: UPDRS off score correlates negatively (*p* < 0.01 corrected) with FA in the right splenium and forceps major in the 30 × 2 dataset, shown overlaid on an MNI152 template and the mean FA skeleton (green).

In the 30 × 2 dataset, as the severity of patients' motor features (UPDRS off medication score) increased, FA decreased in the right splenium and right forceps major (*p* < 0.01). No correlations with UPDRS score at *p* < 0.01 were identified in the 12 × 5 dataset for FA, or in either of the two datasets for MD (see Fig. 2).

### Executive function

In both PD patients and controls, we found that as phonemic fluency score increased, FA increased (*p* < 0.01) and MD decreased (*p* < 0.01) in prefrontal white matter and the anterior corpus callosum. Both 12 × 5 and 30 × 2 datasets additionally identified correlations between phonemic fluency score and FA in the right internal capsule, and the 12 × 5 dataset showed further correlations between phonemic fluency and FA in the external capsules. Table 3 lists all tracts in which FA or MD correlated with phonemic fluency scores in PD patients and controls.

### Voxel-based analyses

At a significance threshold of *p* < 0.01 corrected for multiple comparisons, there were no significant clusters of reduced FA or increased MD in PD patients. However, at a more liberal exploratory statistical threshold of *p* < 0.001 (uncorrected), we observed a similar pattern of white matter alterations to that identified by the TBSS analysis: namely that both the 12 × 5 and 30 × 2 datasets identified reduced FA in PD patients in prefrontal white matter, parietal white matter, and the corpus callosum; and increased MD in prefrontal and parietal white matter (see Fig. 4). As in the TBSS analysis, at this lower statistical threshold the 30 × 2 dataset identified more extensive reductions in FA, including temporal lobe white matter, the left external capsule, and the left cerebellum; whilst the 12 × 5 dataset additionally identified increased MD in temporal lobe white matter, the corpus callosum, and the brainstem.

## Discussion

There are extensive changes in cerebral white matter in PD. We found that early to mid stage (Hoehn and Yahr 1–3) non-demented PD patients have both reduced FA and increased MD across several regions of cerebral white matter. These areas include major white matter tracts in the frontal and parietal lobes. Although the changes in FA and MD had a similar spatial distribution, the dataset acquired with more gradient directions appeared to be more sensitive to reductions in FA, whilst the dataset acquired with more *b* values appeared to be more sensitive to increases in MD (although the direct comparison between the two datasets was not significant). Whilst TBSS identified these widespread alterations in PD white matter microstructure at an appropriately corrected statistical threshold, a voxel-based approach was successful only when a more liberal uncorrected threshold was used. With regards to the impact of white matter pathology on cognition, FA and MD in prefrontal white matter correlated with phonemic fluency, an index of executive function.

Although previous region-of-interest diffusion studies of PD have shown reduced FA in specified subcortical structures and cerebral white matter tracts (Chan et al., 2007; Gattellaro et al., 2009; Modrego et al., 2011; Peran et al., 2010; Rolheiser et al., 2011; Vaillancourt et al., 2009), our results demonstrate more spatially extensive white matter pathology in PD than previously identified in these studies. We have replicated previous findings using whole-brain analysis methods (Hattori et al., 2012; Ibarretxe-Bilbao et al., 2010; Karagulle Kendi et al., 2008; Lee et al., 2010b; Zhan et al., 2012; Zhang et al., 2011), finding reduced FA in the gyrus rectus (olfactory tract), prefrontal white matter, and the corticospinal tract. Here, we also identified reductions in FA in prefrontal, parietal and temporal lobe white matter, throughout the length of the corpus callosum, and in the mid- and superior corticospinal tract. Increases in MD, similarly a marker of white matter pathology, were found in prefrontal, parietal and temporal white matter, the corpus callosum, in the mid- and superior corticospinal tract, and the internal and external capsules. Together, our results suggest that many regions of cerebral white matter are affected in PD, even in early to mid motor stage patients.

It is difficult to make specific inferences as to the underlying axonal or myelin pathology related to changes in FA and MD using DTI alone. It has been suggested that changes in the first tensor eigenvalue, or axial diffusivity (AD), might be related to axonal pathology (such as reduced axonal density or calibre), whilst changes in the second and third tensor eigenvalues, or radial diffusivity (RD), might be related to myelin pathology (Song et al., 2002). We did indeed find increases in both the AD and RD at *p* < 0.01 in many of the regions of white matter identified in the FA and MD statistics as showing pathology in PD patients (see Supplementary information). However, Wheeler-Kingshott and Cercignani (2009) argue that increases in AD or RD should not be over-interpreted for evidence of specific axonal or myelin pathology. Therefore we do not draw specific conclusions as to the particular nature of the pathology underlying the FA and MD changes in PD identified here.

It is well established that a fronto-striatal dysexecutive syndrome, accompanied by grey matter pathology affecting fronto-striatal circuits, can be present from the early stages of PD (Foltynie et al., 2004; Lee et al., 2010a; Nishio et al., 2010; Owen et al., 1992, 1993; Song et al., 2011; Williams-Gray et al., 2007, 2009). Here, we have demonstrated corresponding evidence for fronto-striatal white matter pathology, with reduced FA and increased MD in prefrontal white matter tracts, and increased MD in the external capsules (one of the principal white matter tracts connecting the prefrontal cortex and the striatum).

Phonemic fluency was not impaired in the patient group as a whole. However, we observed correlations between FA and MD in prefrontal white matter tracts and subjects' phonemic fluency scores. FA in the external capsules also correlated with phonemic fluency. Taken together, these results suggest that fronto-striatal white matter tract microstructure, considerably altered in PD, is related to executive function. Pathology in fronto-striatal white matter may be a potential contributing factor to the PD dysexecutive syndrome.

Patients' UPDRS scores correlated with FA in the right splenium of the corpus callosum. However, that there were not more extensive correlations with UPDRS scores indicates that the widespread pathology identified by the main group difference is present across a range of motor severities. We note however that our study included only patients in an early to mid stage of the disease, using the Hoehn and Yahr staging system that is based on motor signs, mobility and balance. The patients varied widely in their duration of illness, and cognitive function, which are not reflected in the staging system.

We asked whether two diffusion imaging protocols would differ in their sensitivity to white matter tract alterations in PD: one prioritising gradient directions, the other having fewer directions but a larger range of *b* values in the same total scan time (10 min). We did not compare multiple acquisitions using a single *b* value with multiple acquisitions using a range of *b* values. Given the possibility of larger motion artefacts in the patient group, we also considered whether improved estimation of diffusivity per direction, derived from multiple *b* values, was important. In fact, PD patients moved no more during either of the two 10-minute diffusion image acquisitions than controls. Thus, this motivation for a diffusion imaging protocol with a large number of *b* values was found to be unsubstantiated.

The two protocols revealed contrasting benefits with regards to detection of group differences in FA and MD. Previous work with simulated data suggested that a 12 × 5 protocol with multiple *b* values might benefit accurate estimation of MD, whilst a 30 × 2 protocol with a larger number of gradient directions might improve the estimation of FA (Correia et al., 2009). The simulations also indicated that for tissues with a range of diffusivities (as in the brain), and lower signal to noise, a diffusion imaging protocol with multiple *b* values is likely to give more accurate measures of MD (Correia et al., 2009). Although the estimation of FA may also benefit from multiple *b* values, the number of gradient directions was found to be more important. A larger number of gradient directions give greater angular resolution, which facilitates the calculation of relative diffusion in orthogonal axonal and radial orientations, underlying the estimation of FA. Whilst we cannot infer greater accuracy of estimation of FA or MD, we found that the two sequences differed in sensitivity to white matter pathology in PD: the 12 × 5 dataset identified more widespread increases in MD, whilst in contrast, the 30 × 2 dataset identified more widespread reductions in FA. Despite these differences between protocols, it is striking that the regional changes in FA identified by the 30 × 2 dataset and in MD by the 12 × 5 dataset overlap considerably (see Fig. 1). This suggests that given an appropriate diffusion imaging protocol, similar regions of white matter pathology can be identified in both FA and MD maps.

**Fig. 3:**
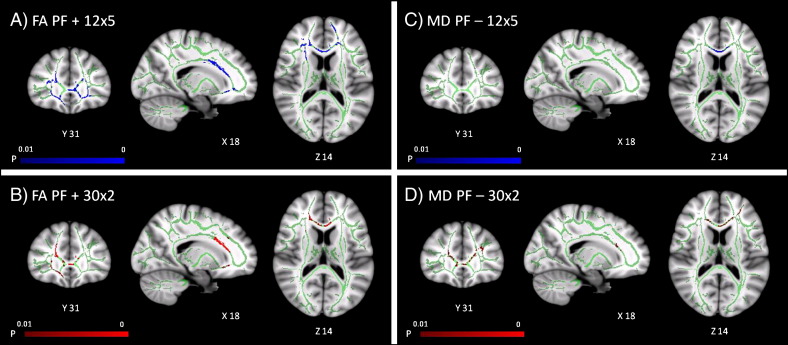
TBSS: regions where phonemic fluency correlates positively (*p* < 0.01 corrected) with FA in A) the 12 × 5 and B) 30 × 2 datasets; and correlates negatively (*p* < 0.01 corrected) with MD in C) the 12 × 5 and D) 30 × 2 datasets, shown overlaid on an MNI152 template and the mean FA skeleton (green).

**Fig. 4:**
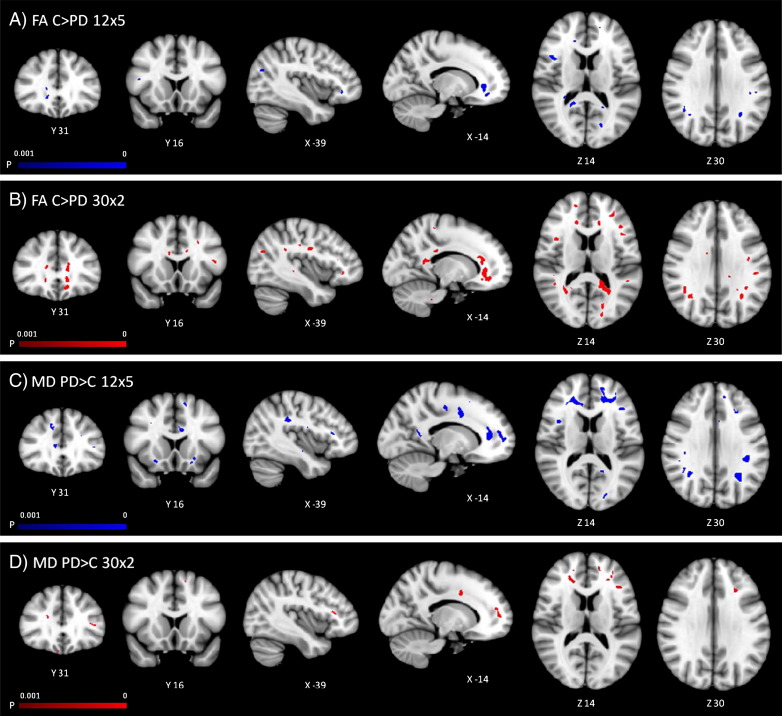
Voxel-based analysis: Regions of reduced FA in PD patients in A) the 12 × 5 dataset and B) 30 × 2 dataset, and increased MD in C) the 12 × 5 and D) 30 × 2 datasets, shown at a liberal threshold of *p* < 0.001 uncorrected with cluster size threshold > 50 voxels.

Despite the apparent differences in thresholded statistics, the two imaging protocols were not significantly different from each other in a direct comparison (as there were no significant interactions between group and diffusion imaging protocol for FA or MD). The difference between the two imaging protocols in detection of group differences in FA and MD must therefore be viewed cautiously (Nieuwenhuis et al., 2011). In addition, the correlations between phonemic fluency and FA or MD do not suggest that the 30 × 2 protocol is more sensitive to FA correlations with phonemic fluency, or the 12 × 5 protocol to MD correlations. In fact, a small number of skeleton voxels showed the opposite relationship for correlation with phonemic fluency. However, the difference in sensitivity to the phonemic fluency correlations for the two protocols is relatively small (see Fig. 3 and Table 3), approximately 1000–2500 voxels. This contrasts with the marked difference in sensitivity to the main group difference for the two protocols (see Fig. 1 and Table 2), at approximately 14,000 voxels. The different sensitivity of the two protocols to FA and MD correlations with fluency must be interpreted with caution, given the lack of significance of the direct comparisons, the comparable fluency between groups, and that the regions with significant correlations overlap rather than segregate. Overall, we therefore place greater emphasis on commonality of these correlations with fluency, and the greater sensitivity of group effects to FA through more directions, rather than the marginal advantage for correlations with FA through more *b* values.

TBSS was successful in identifying widespread group differences in FA and MD between PD patients and controls, and in identifying associations between cognition and white matter microstructure. A voxel-based approach, however, was less sensitive, and identified group differences only at a more liberal statistical threshold with no correction for multiple comparisons. Bearing in mind this caveat, the voxel-based analysis showed a highly similar pattern of white matter pathology in PD to the TBSS analysis, namely reductions in FA and increases in MD in the prefrontal, parietal and temporal white matter, the corpus callosum, external capsules, and the corticospinal tract. As in the TBSS analysis, voxel-based methods showed more widespread FA reductions in the 30 × 2 dataset, whilst the 12 × 5 dataset showed more widespread MD increases. The corroboration of the TBSS and voxel-based results suggests that the TBSS results were not obtained merely as a consequence of mis-registration artefacts.

Our results suggest that TBSS can be a more sensitive method than VBM when comparing diffusion measures in PD patients and controls. We used the appropriate standard statistical correction methods for TBSS and VBM respectively, to ensure our procedures were commensurate with common practice. It is therefore difficult to establish whether the benefit of TBSS is obtained from more accurate alignment of the centre of white matter tracts through the registration and skeletonisation process, from non-parametric statistics, from the specific threshold-free cluster enhancement method, or a combination of these differences. However, our aim was to investigate whether the TBSS or VBM procedures as a whole differed in sensitivity to white matter pathology in PD. We hope that in doing so, our findings will be of benefit to other members of the neuroimaging community conducting similar patient–control comparisons, as well as stimulating further research into the critical factors underlying sensitive group comparisons of DTI data.

Despite the demonstration of considerable white matter pathology in PD, there are some limitations to our study. Firstly, diffusion data were collected as part of a larger PD study in which patients were scanned once whilst taking their normal dopaminergic medication, and once after medication delay. However, it is unlikely that the medication contributes to the differences in diffusion measures, for several reasons. The pharmacological action of these drugs makes an effect on DTI data a priori less likely than a primary effect of disease. Importantly, post hoc analysis confirmed that the l-dopa dose equivalent did not correlate with FA or MD. In addition, the two diffusion datasets were counterbalanced for patients being on- or off-medication, so the contribution of medication status to the protocol differences observed for FA and MD was minimised. A second limitation is that our scan order was not fully counterbalanced with regards to the two diffusion imaging protocols. However, measurable differences from significant degeneration are unlikely to have occurred during our mean scan time interval of 3 weeks. Moreover, the differential sensitivities of the two datasets to FA and MD cannot be explained by disease progression during this interval. Thirdly, although the numbers of our subjects (14 PD patients, 15 controls) are comparable to several other DTI studies of PD, some studies have used much larger groups of subjects, reducing type II error (Chan et al., 2007; Hattori et al., 2012; Peran et al., 2010; Zhang et al., 2011). Nevertheless, we have controlled tightly for type I error, and demonstrated large group differences between PD patients and controls in both the 12 × 5 and 30 × 2 datasets with fourteen PD patients and fifteen controls.

It should also be noted that whereas we followed currently standard practices in the acquisition, pre-processing and analysis of the data, for both TBSS and VBM, these methods make a number of assumptions and caveats (see review by Jones and Cercignani, 2010). For example, we used a linear estimation of the diffusion tensor, and assume Gaussian noise. This tensor fit may be appropriate at lower *b* values (1200 s/mm^2^ or less, as in this study) but not at high *b* values (3000 s/mm^2^ or more, Jones and Basser, 2004). Such higher *b* values may become more common with the development of high-angular-resolution diffusion imaging for enhanced tractography. In addition, a common objection to whole-brain analysis methods of diffusion measures is that as a result of the registration process, one cannot be certain that the location identified by voxelwise statistics as showing a significant group difference corresponds to the same anatomical region, or indeed, to white matter at all, in each subject's native space. To address this concern we used the TBSS function “deproject” to validate the registration process by deprojecting any generated statistic images showing regions of significance back to individual native space, and visually inspecting each deprojected statistic image. This confirmed that all regions showing significant group differences or associations with cognitive or motor function in the TBSS analysis corresponded to white matter in each subject.

Future work might examine the impact that the widespread white matter pathology in PD identified here has upon network function of the brain. The diversity of motor and cognitive features associated with PD is likely linked to a combination of both local grey matter dysfunction, and white matter connectional pathology. Such a combination of pathology may be associated with the impairment in PD of fronto-striatal networks essential for normal cognitive and motor function. Future approaches could jointly characterise the pathology of such networks structurally and functionally in PD, for example by combining tractography and effective connectivity analyses.

## Conclusions

We found that white matter pathology is widespread in PD, but is most marked in the frontal lobes. Furthermore, white matter pathology in fronto-striatal networks may be a contributing factor to the PD dysexecutive syndrome. Our comparison of different acquisition protocols with which to collect diffusion imaging data in patients, and evidence that TBSS is more sensitive than voxel-based analysis, will be of use to researchers wishing to conduct future DTI studies.

## Funding

This work was supported by the Wellcome Trust [088324 to JBR, LEH, EA]; the Medical Research Council UK [MMC; Doctoral Training Award to CLR; and MC_A060_5PQ30]; Parkinson's UK [RAB]; and the National Institute for Health Research Cambridge Comprehensive Biomedical Research Centre.

## Figures and Tables

**Table 1:** Demographic and neuropsychological evaluation information for PD patients and controls, and disease severity information for PD patients. Mean (range). UPDRS = Unified Parkinson's Disease Rating Scale (subscale III).

	PD patients (*n* = 14)	Controls (*n* = 15)	Statistic *p*-value
Male/female	7/7	10/5	0.36^a^
Age	65 (51:78)	64 (50:75)	0.71^b^
Phonemic fluency score	14.57 (11:24)	15 (7:21)	0.81^b^
Mini-mental state examination	28.36 (26:30)	29 (26:30)	0.14^b^
Disease duration (years)	10 (4:20)	–	–
UPDRS on	10 (3:19)	–	–
UPDRS off	20 (14:32)	–	–
Hoehn and Yahr on	1.9 (1:3)	–	–
Hoehn and Yahr off	2.1 (1.5:3)	–	–
L-dopa equivalent daily dose (mg)	1388 (540:2160)	–	–

a *x*^2^ test.

b *t*-test.

**Table 2:** Tracts with reduced FA or increased MD (*p* < 0.01 corrected) in PD patients in the 12 × 5 and 30 × 2 datasets, localised according to the John Hopkins University ICBM-DTI-81 White Matter Labels and John Hopkins University White Matter Tractography atlases in FSL (B = bilateral; L = left hemisphere, R = right hemisphere).

Tract	FA		MD	
12 × 5	30 × 2	12 × 5	30 × 2
Forceps minor	B	B	B	B
Corpus callosum	B	B	B	B
Forceps major	B	B	L	–
Anterior thalamic radiation	R	B	B	B
Uncinate fasciculus	B	B	B	B
Inferior fronto-occipital fasciculus	B	B	B	B
Internal capsule	–	–	L	–
External capsule	–	–	B	–
Corticospinal tract	R	B	L	–
Superior longitudinal fasciculus	R	B	B	–
Inferior longitudinal fasciculus	–	B	L	–
Total *n* of voxels at *p* < 0.01	6395	20771	15403	1617

**Table 3:** Tracts with correlations between increased FA or decreased MD and phonemic fluency (*p* < 0.01 corrected) in PD patients and controls, in the 12 × 5 and 30 × 2 datasets, localised according to the John Hopkins University ICBM-DTI-81 White Matter Labels and John Hopkins University White Matter Tractography atlases in FSL (B = bilateral; L = left hemisphere, R = right hemisphere).

Tract	FA		MD	
12 × 5	30 × 2	12 × 5	30 × 2
Forceps minor	B	B	–	B
Corpus callosum	B	B	B	B
Forceps major	–	–	–	–
Anterior thalamic radiation	B	R	–	B
Uncinate fasciculus	B	R	L	B
Inferior fronto-occipital fasciculus	B	R	L	B
Internal capsule	R	R	–	–
External capsule	B	–	–	–
Corticospinal tract	–	–	–	–
Superior longitudinal fasciculus	R	–	L	L
Inferior longitudinal fasciculus	–	–	–	–
Total *n* of voxels at *p* < 0.01	4426	1979	1303	2060
