# Supplementary material for: White matter pathology in Parkinson's disease: The effect of imaging protocol differences and relevance to executive function
Source: Neuroimage. 2012 Sep;62(3-2):1675–84. doi: 10.1016/j.neuroimage.2012.06.012 (PMC3413883; doi:10.1016/j.neuroimage.2012.06.012)
Supplement: Supplementary file 1 — Supplementary material. [file mmc1.doc]

**Supplementary Information**

**White matter pathology in Parkinson’s disease: the effect of imaging protocol differences and relevance to executive function**

Charlotte L. Rae^a^,, Marta M. Correia^a^, Ellemarije Altena^b^, Laura E. Hughes^a,b^, Roger A. Barker^b,c^, James B. Rowe^a,b,d^

^a^ MRC Cognition and Brain Sciences Unit, Cambridge, CB2 7EF, UK

^b^ Department of Clinical Neurosciences, University of Cambridge, Cambridge, CB2 2QQ, UK

^c^ Cambridge Centre for Brain Repair, University of Cambridge, CB2 0PY, UK

^d^ Behavioural and Clinical Neuroscience Institute, University of Cambridge, Cambridge, CB2 3EB, UK

Table S1. PD patients’ medication information. H&Y=Hoehn & Yahr; UPDRS=Unified Parkinson’s Disease Rating Scale; LEDD=levodopa equivalent daily dose; mg=milligrams. †Calculated according to Williams Gray et al. (2007) *J Neurosci*. *mg/day of levodopa in combined levodopa/carbidopa medications (Sinemet, Madopar, Stalevo).

| **Subject** | **Sex** | **Age** | **Disease duration** | **H&Y (on)** | **UPDRS on** | **UPDRS off** | **LEDD**† | **PD medications (mg/day)** * | **Additional medications** |
| --- | --- | --- | --- | --- | --- | --- | --- | --- | --- |
| 1 | M | 57 | 11 | 1 | 6 | 18 | 1340 | Pramipexole 2.1  Sinemet Plus 500 | Fluoxetine |
| 2 | M | 71 | 5 | 1 | 10 | 17 | 900 | Ropinerole 15  Sinemet 300 | Simvastatin |
| 3 | M | 60 | 12 | 2 | 9 | 22 | 1170 | Amantadine 200  Madopar 150  Pramipexole 2.55 | - |
| 4 | M | 51 | 5 | 2 | 7 | 23 | 1080 | Madopar 200  Pramipexole 2.1  Selegiline 10 | - |
| 5 | F | 69 | 8 | 2 | 18 | 19 | 1780 | Amantadine 100  Cobeneldopa 500  Madopar 50  Ropinerole 16  Stalevo 800 | Clonazepam  Fluoxetine  Indepamide  Levothyroxine sodium  Omeprazole |
| 6 | F | 73 | 7 | 2 | 11 | 16 | 1160 | Ropinerole 24  Sinemet 200 | Citalopram  Modafinil |
| 7 | M | 73 | 11 | 1.5 | 3 | 24 | 1380 | Ropinerole 21  Stalevo 450 | Amitriptyline  Amlodipine  Perindopril  Simvastatin |
| 8 | F | 53 | 14 | 2 | 19 | 32 | 1740 | Amantadine 200  Pramipexole 3.15  Stalevo 400 | - |
| 9 | F | 63 | 13 | 2 | 15 | 18 | 840 | Entacapone 600  Ropinerole 12  Sinemet Plus 300 | - |
| 10 | F | 64 | 8 | 1.5 | 7 | 18 | 2056 | Pramipexole 2.64  Sinemet 400  Sinemet Plus 600 | Amitriptyline  Candesartin cilexetil  Co-amilozide |
| 11 | M | 63 | 12 | 2 | 12 | 14 | 2160 | Amantadine 400  Cobeneldopa 900  Pramipexole 3.15 | Propanolol  Sildanefil citrate |
| 12 | F | 74 | 20 | 2 | 6 | 18 | 1560 | Rasagiline 1  Ropinerole 24  Sinemet Plus 500 | Amitriptyline  Bisoprolol fumarate  Levothyroxine  Ramipiril |
| 13 | M | 78 | 14 | 3 | 17 | 16 | 1720 | Rasagiline 1  Ropinerole 16  Stalevo 750 | Amlodipine  Tamsulosin hydrochloride |
| 14 | F | 64 | 4 | 2 | 4 | 20 | 510 | Madopar 125  Stalevo 300 | Liviol  Venlafaxine |

Table S2. Mean total amount of subject movement (mm) by PD patients and controls during each imaging sequence.

| **Imaging protocol** |  | **Mean total movement (mm)** | **SD** | **N** |
| --- | --- | --- | --- | --- |
| 12x5 | Control | 23.62 | 4.00 | 15 |
|  | PD | 22.32 | 5.91 | 14 |
|  | Total | 22.99 | 4.97 | 29 |
| 30x2 | Control | 23.08 | 5.96 | 15 |
|  | PD | 23.66 | 5.23 | 14 |
|  | Total | 23.36 | 5.53 | 29 |

Table S3. Number of significant voxels in each TBSS statistic image. UPDRS=Unified Parkinson’s Disease Rating Scale; PF=phonemic fluency; FA=fractional anistotropy; MD=mean diffusivity.

| **Contrast** | **Dataset** | ***p* value** | **n voxels** |
| --- | --- | --- | --- |
| FA |  |  |  |
| C>PD | 12x5 | <0.01 | 6395 |
|  | 30x2 |  | 20771 |
| UPDRS | 12x5 | <0.01 | 0 |
|  | 30x2 |  | 85 |
| PF | 12x5 | <0.01 | 4426 |
|  | 30x2 |  | 1979 |
| MD |  |  |  |
| PD>C | 12x5 | <0.01 | 15403 |
|  | 30x2 |  | 1617 |
| UPDRS | 12x5 | <0.01 | 0 |
|  | 30x2 |  | 0 |
| PF | 12x5 | <0.01 | 1303 |
|  | 30x2 |  | 2060 |

**
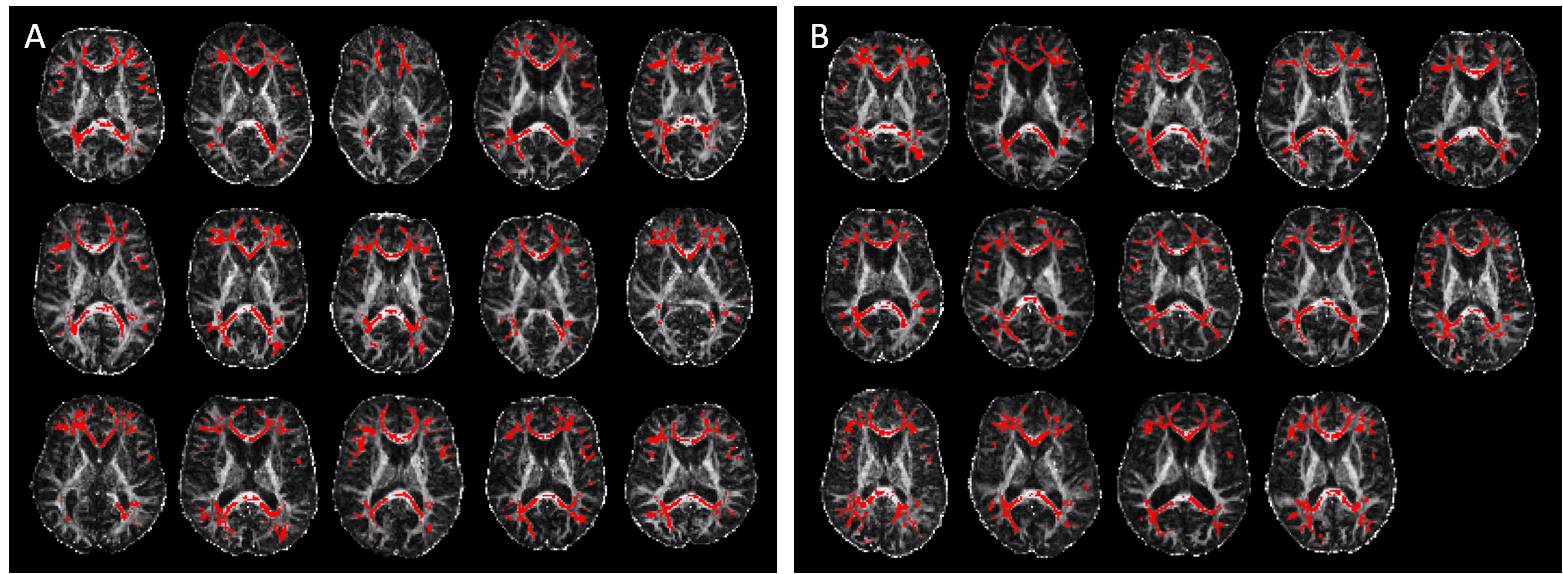
**

Figure S1. TBSS Deproject. Sample TBSS statistic (FA C>PD 30x2) deprojected back into each subject’s native space on a mid axial slice, demonstrating accurate initial registration to the study-specific template for A) controls, and B) PD patients.


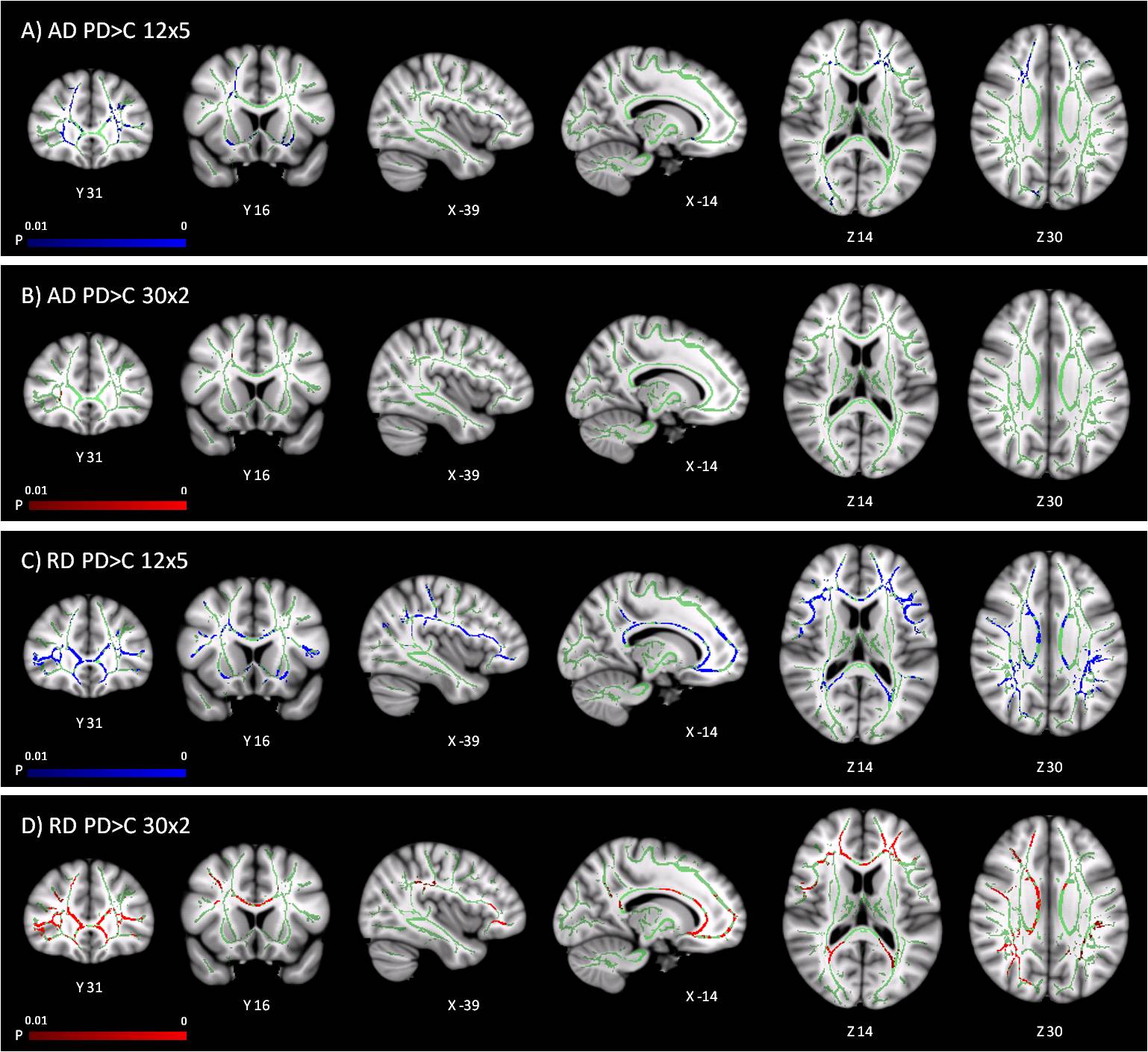


Figure S2. Regions of increased axial diffusivity (AD) (*p*<0.01 corrected) in PD patients in A) the 12x5 dataset, and B) 30x2 dataset, and increased radial diffusivity (RD) (*p*<0.01 corrected) in C) the 12x5 and D) 30x2 datasets. TBSS results are shown overlaid on an MNI152 template and the mean FA skeleton (green).
